# Task Planning of Multiple Unmanned Aerial Vehicles Based on Minimum Cost and Maximum Flow

**DOI:** 10.3390/s25051605

**Published:** 2025-03-05

**Authors:** Xiaodong Shi, Xiangping Zhai, Rui Wang, Yi Le, Shuang Fu, Ningzhong Liu

**Affiliations:** 1 College of Computer Science and Technology/Artifical Intelligence, Nanjing University of Aeronautics and Astronautics, Nanjing 211106, China; shixiaodong@nuaa.edu.cn (X.S.); ningzhongliu@nuaa.edu.cn (N.L.); 2 Nanjing Research Institute of Electronic Engineering, Nanjing 210007, China; lyy2023@whu.edu.cn; 3 Key Laboratory of Brain-Machine Intelligence Technology, Ministry of Education, Nanjing 211106, China; 4 Collaborative Innovation Center of Novel Software Technology and Industrialization, Nanjing 210023, China

**Keywords:** UAV delivery, vehicle routing problem, minimum-cost, maximum-flow model, resource tree, task planning

## Abstract

With the rapid development of UAV technology, UAV delivery has gained attention for its potential to reduce labor costs. However, limitations in load capacity and energy restrict UAVs’ distribution capabilities. This paper proposes a cooperative delivery scheme combining traditional trucks and UAVs to extend UAV coverage and improve delivery completion rates. For densely distributed depots in wide-area regions, we develop algorithms for task allocation and path planning in a truck-independent UAV system. Specifically, a minimum-cost, maximum-flow model is constructed to obtain sub-paths covering all delivery tasks, and resource tree-based algorithms are used to construct global paths for UAVs and trucks. Simulation results show that our algorithms reduce total energy consumption by 11.53% and 9.15% under different task points, which suggests that our proposed method can significantly enhance delivery efficiency, offering a promising solution for future logistics operations.

## 1. Introduction

In recent years, Unmanned Aerial Vehicle (UAV) technology has made significant advancements, achieving breakthroughs in key performance metrics such as flight stability, endurance, and payload capacity [1,2,3]. The continuous reduction in the cost of small and lightweight UAVs has rapidly expanded their civilian applications beyond military reconnaissance and aerial photography. This cost reduction has laid a technical and economic foundation for UAVs to enter the field of logistics and delivery [4,5]. UAVs are unaffected by ground traffic conditions, can fly in straight lines, and can quickly traverse congested areas. Particularly for urgent small packages, UAVs can significantly reduce delivery time, enabling same-city or even short-distance intercity express delivery services, thereby enhancing customer satisfaction [6,7,8]. Additionally, in remote areas where truck delivery costs are high and single trips may face the challenge of returning empty, UAVs can provide flexible on-demand delivery. This reduces unnecessary labor, vehicle wear, and fuel costs, ultimately optimizing the logistics cost structure over the long term.

However, due to limitations in endurance, payload capacity, and flight range, relying solely on UAVs for all deliveries may not be optimal [9,10]. Integrating UAVs and trucks for collaborative delivery can effectively address the challenges faced by using either UAVs or trucks alone. First, trucks, with their longer travel range and greater load capacity, can serve as mobile depots for storing packages and providing energy for UAVs. This role significantly extends the effective delivery range of UAVs [11,12]. Second, UAVs are unable to deliver packages that exceed their payload capacity or are too large in volume. Trucks can handle these packages, ensuring higher delivery completion rates when working collaboratively with UAVs. Furthermore, UAVs are not restricted by ground traffic conditions and perform better in areas with complex road conditions that are difficult for trucks to access. By collaborating, UAVs and trucks integrate their respective advantages. Trucks serve as mobile depots and long-distance transportation tools, carrying multiple UAVs and large quantities of packages over long distances, while UAVs handle last-mile precise delivery. Each vehicle fulfills its role to maximize delivery efficiency [13,14].

Most existing research on the collaborative distribution of trucks and UAVs adopts the single-UAV and single-vehicle collaborative distribution model. Unfortunately, UAVs can typically deliver to only one or two task points at a time. Moreover, due to the numerous loading and unloading procedures for materials, the efficiency of a single delivery is low, resulting in relatively long times to complete the distribution tasks. Compared to the single-UAV and single-vehicle collaboration and considering the small size and low load capacity of small UAVs, this paper proposes the adoption of a multi-vehicle and multi-UAV joint distribution method to improve distribution efficiency. By taking the depot as the supply point for UAV packages and energy, UAVs can take off and land at different locations, expanding their delivery range and achieving the goal of distribution in wide-area scenarios. In this paper, we mainly study how independent UAVs and trucks can jointly deliver packages in a wide-area scenario with densely distributed depots, where UAVs can replenish energy in a timely manner. Our main contributions are outlined as follows:

- We propose a novel task-planning framework for a truck–UAV system, specifically targeting the minimization of system energy consumption in wide-area scenarios with dense supply-point depots. Our approach uniquely addresses the challenges of handling overweight tasks and tasks beyond UAV delivery ranges by strategically assigning these to trucks, thereby optimizing the overall system efficiency and energy utilization.
- Recognizing the load and energy limitations of UAVs, we develop a sophisticated modeling approach by constructing minimum-cost, maximum-flow models for both UAVs and trucks. This allows us to derive optimal sub-paths that cover all delivery tasks with minimal energy consumption. We further enhance this approach by introducing a depot–task set–depot combination strategy, which enables efficient path construction using a resource tree-based algorithm for UAVs and an insertion method for trucks. This integrated method not only ensures comprehensive task coverage but also achieves significant energy savings compared to traditional approaches.
- We conduct comprehensive simulation experiments to validate the feasibility and effectiveness of our algorithms. The results demonstrate that our proposed method outperforms existing solutions in terms of energy efficiency and task completion rates, highlighting the superiority of our approach. Our contributions go beyond mere simulation validation by providing a robust and innovative solution to the complex task-planning problem in truck–UAV systems.

The rest of this paper is organized as follows. Section 2 introduces related works. Section 3 details the system model. Section 4 details the algorithm proposed in this paper. In Section 5, we carry out numerical experiments and obtain the simulation results. Section 6 concludes our work.

## 2. Related Work

Collaboration between trucks and UAVs in delivery can reduce truck energy consumption, allow for simultaneous delivery operations, and significantly improve overall delivery efficiency. Consequently, researchers have been exploring more cost-effective and faster collaborative delivery schemes. N. Agatz et al. [15] proposed the classical Traveling Salesman Problem with Drone (TSP-D) delivery model, where a truck carries a UAV for delivery. While the truck serves one customer, the UAV delivers to other customers within its range, then returns to the truck to reload packages. The authors formulated this problem as a mixed-integer programming model and solved it using a combination of local search and dynamic programming. First, the truck’s route is calculated using the TSP method. Then, a greedy heuristic algorithm is applied to partition the route and assign tasks to the truck and UAV. Finally, local search is employed to optimize the routes of both.

Murray and Chu [16] introduced two new models: FSTSP (Flying Sidekick TSP) and PDSTP (Parallel Drone Scheduling TSP). FSTSP is designed for scenarios where the depot is far from the customers and the UAV cannot complete deliveries independently. The goal is to minimize overall delivery time, and the authors developed a mathematical model and heuristic search algorithm for this purpose. Conversely, the PDSTP model is applicable when the depot is close to the customers and most customers are within the UAV’s delivery range. In this case, the UAV and truck independently pick up and deliver packages.

In [17], the authors proposed a decomposition-based iterative algorithm aimed at minimizing delivery time and reducing system energy consumption. The first phase determines the truck route and assigns customers to the UAV for delivery. The second phase solves a mixed-integer linear programming model to optimize the UAV’s flight paths based on the results of the first phase. Starting with the shortest truck route, the algorithm iteratively improves task allocation and route planning for both the truck and UAV.

Most previous algorithms restrict UAVs to delivering a single package per trip. However, Y. Liu et al. [18] proposed that UAVs could carry multiple packages between the truck and customers, provided that the payload and energy constraints are satisfied, aiming to minimize system energy consumption. Their study considered the impact of package weight on energy consumption and derived an energy consumption formula as a function of total package weight. A two-phase route construction method was employed: an initial solution obtained by combining the nearest-neighbor and savings algorithms and a hybrid optimization algorithm combining simulated annealing and tabu search to refine the solution, yielding a collaborative delivery scheme. In [19], to address the TSP-D problem and reduce delivery time and energy consumption, the authors proposed a novel method tailored for a cutting-plane algorithm. Two mixed-integer linear programming (MILP) models, MILP-A and MILP-B, were constructed. The MILP models were generated using existing methods and inequality-driven mechanisms, followed by a branch-and-cut algorithm that produced MILP-BC-VI. Experimental results demonstrated that this method performs excellently on various benchmark instances, enabling rapid processing of small-scale customer cases and solving larger-scale instances within one hour. In [20], the authors proposed a new two-stage MIP method and an exact-solution approach based on full decomposition to tackle the TSP-D model. The problem was formulated as a mixed-integer programming (MIP) model and divided into two decision-making stages: first, a subset of customers is selected for truck delivery and ordered; then, UAV routes are planned for the remaining customers. Structural properties of the optimal TSP-D solution were further optimized to improve solution performance.

In [21], the authors investigated the problem of multi-drop delivery coordinated by trucks and UAVs, proposing a heuristic approach to optimize route planning. The study focused on how to coordinate task allocation and route design between trucks and UAVs to maximize delivery efficiency. In [22], the authors studied the synchronized routing problem of trucks and UAVs, addressing the challenge of achieving efficient collaboration between the two in package delivery. They proposed a multi-objective optimization model aimed at minimizing delivery time and cost while enhancing system efficiency. By designing a Multi-Objective Ant Colony Optimization (MOACO) algorithm, the model effectively handles task allocation, route planning, and UAV endurance constraints. In [23], the authors investigated a hybrid truck–UAV collaborative delivery problem where UAVs can operate either independently or be deployed by trucks at task locations. They proposed a strategy for route planning and scheduling to optimize task allocation, route design, and UAV energy usage. By employing a mixed-integer linear programming (MILP) model and heuristic algorithms, the method effectively addresses the complex constraints of hybrid delivery, significantly improving task completion efficiency. In [24], the authors focused on the problem of truck–UAV collaboration for urban emergency response, proposing a dynamic collaboration and integrated route-planning method. The study emphasized delivery tasks in disaster management scenarios, designing a multi-objective optimization model that incorporates route flexibility, delay minimization, and resource optimization. Experimental results demonstrated that the proposed method offers significant advantages in handling emergencies and resource-constrained scenarios.

However, existing research still has some limitations. First, most studies have focused on a single scenario or specific types of tasks, lacking a systematic exploration of task planning in wide-area scenarios with densely distributed supply points. Second, existing methods often fail to optimize task allocation and path planning simultaneously when dealing with UAV range and payload constraints, resulting in higher system energy consumption. Moreover, there is a lack of effective solutions for handling tasks beyond UAV delivery ranges or overweight tasks, making it difficult to achieve efficient collaboration between trucks and UAVs. To address these issues, this paper proposes a novel task-planning framework targeting wide-area scenarios with densely distributed supply points, with the goal of minimizing system energy consumption. Our approach assigns overweight tasks and tasks beyond UAV delivery ranges to trucks, thereby optimizing overall system efficiency and energy utilization. Additionally, we construct minimum-cost, maximum-flow models for both UAVs and trucks and introduce a resource tree-based algorithm and insertion method to achieve efficient path planning. Comprehensive simulation experiments demonstrate the feasibility and effectiveness of our algorithms. The results show that our method outperforms existing solutions in terms of energy efficiency and task completion rates, providing an innovative and robust solution to the complex task-planning problem in truck–UAV systems.

## 3. System Model

**Figure 1:**
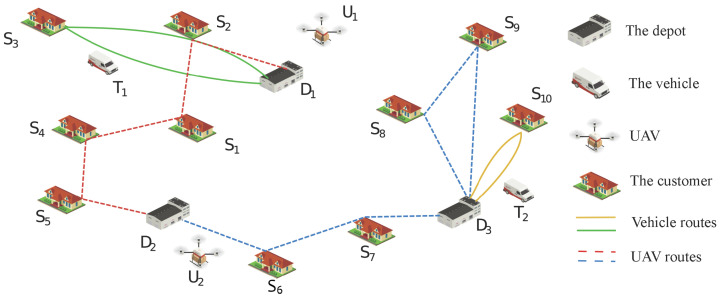
Illustration of the truck-independent UAV system.

As shown in Figure 1, the scenario we are studying is a wide-area scenario with a sufficient number of depots acting as supply stations. The scenario includes multiple depots, several trucks, a group of customers, and multiple UAVs. In this scenario, the multi-UAV logistics mission-planning problem can be described as follows: In a known environment, there are *Q* depots that can serve as supply stations to replenish energy and packages for UAVs and trucks and can also serve as the starting or ending points for each UAV and truck delivery mission; there are *F* customers, who are the task points requiring package delivery; and there are *E* trucks, which are responsible for delivering packages that are overweight and beyond the UAV delivery range. Trucks have load limitations but no driving distance restrictions; there are *N* UAVs, which have load and energy storage limitations. The task is to assign missions to UAVs and trucks and plan their routes under the conditions of the UAVs’ load and energy limitations and the trucks’ load limitations so as to minimize the overall energy consumption of completing all delivery tasks. For any depot taken as the center of a circle, with the radius being half of the maximum flying distance of a UAV, the final result obtained by the intersection of circles drawn based on all depots is the delivery range of the UAVs.

Due to the energy and load limitations of the UAVs, it is impossible for a UAV to complete all tasks in one flight. Therefore, it is necessary to arrange depots in a timely manner for the UAVs to land and replenish energy. This means we need to plan the takeoff and landing depots for the UAVs, as well as the delivery tasks to be completed after takeoff. Excluding task points not within the UAV delivery range, considering task points within the delivery range with package weights exceeding the maximum load of the UAVs, these task points must be delivered by trucks. The set of task points for which UAVs are responsible is denoted as $S_{U}$, and the set of task points for which trucks are responsible is denoted as $S_{T}$. It should be noted that trucks have load limitations and can only carry a certain weight of packages at a time. Therefore, the problem that needs to be studied is how to reasonably allocate tasks to UAVs and trucks without exceeding the limitations and minimizing the overall cost.

We define the set of depots within the task area as $D=\{D_{1},D_{2},\cdots ,D_{Q}\}$, where $D_{q}$ represents the *q*-th depot. We define the set of UAVs participating in the distribution as $U=\{U_{1},U_{2},\cdots ,U_{N}\}$, where $U_{n}$ represents the *n*-th UAV, and we define the maximum flight distance of the UAVs as $L_{max}$ and the maximum load capacity as $M_{max}^{u}$. We define the set of trucks participating in the distribution as $T=\{T_{1},T_{2},\cdots ,T_{E}\}$, where $T_{e}$ represents the *e*-th truck and the maximum load capacity of the truck is $M_{max}^{t}$. We define the set of task points within the task area as $S=\{S_{1},S_{2},\cdots ,S_{F}\}$, where $S_{f}$ represents task point *f* and it is stipulated that each task point can only be executed once. The weights of the packages corresponding to each task point are different. We define $M=\{M_{1},M_{2},\cdots ,M_{F}\}$ as the package weights of each task point, where $M_{F}$ represents the package weight of task point *f*. There are many factors influencing the task allocation of collaborative distribution based on independent UAVs. The important notations are defined in Table 1.

In order to simplify the model, reduce the difficulty of model solution, and ensure maximum consistency with actual UAV delivery, the following reasonable assumptions are made for model construction:

- UAVs do not encounter obstacles during flight.
- The energy consumption of UAVs caused by take-off, landing, and natural factors is ignored.
- The flight speed of UAVs is constant, and the driving speed of transport vehicles is constant.
- All distribution tasks are equally important. In the same environment, there is no priority ranking for package distribution, and the model does not consider the time window for the delivery of materials for the time being.

The specific distribution process is shown in Figure 1, where the solid lines represent the routes of the trucks and the dashed lines represent the flight routes of the UAVs. Suppose a scenario contains three depots and ten task points, as well as two UAVs and two trucks. The initial docking points of UAV $U_{1}$ and $U_{2}$ are at depot $D_{1}$ and $D_{2}$, respectively, and the initial docking points of trucks $T_{1}$ and $T_{2}$ are at $D_{1}$ and $D_{3}$, respectively. UAV $U_{1}$ takes off from depot $D_{1}$, then executes tasks $S_{2}$ to $S_{5}$ along the route before landing at depot $D_{2}$. At the same time, UAV $U_{2}$ takes off from depot $D_{2}$, then executes tasks $S_{6}$ and $S_{7}$ along the route before landing at depot $D_{3}$. After replenishing the power and goods, it takes off again, executes tasks $S_{8}$ and $S_{9}$ along the route, and lands at depot $D_{3}$. Truck $T_{1}$ departs from $D_{1}$, executes all tasks along the route, and finally arrives at depot $D_{1}$. Truck $T_{2}$ departs from $D_{3}$, executes all tasks along the route, and finally arrives at depot $D_{3}$. The purpose of our research is to assign appropriate tasks to UAVs and trucks and plan their routes under the constraints of UAVs and trucks so as to complete all distribution tasks and minimize the final total cost. In addition, some constraints that need to be satisfied for the task-planning problem of the truck–independent UAV system are described as follows:

1. The take-off and landing points of each UAV flight can only be depots, and UAVs cannot dock elsewhere.
2. If a UAV lands at a certain depot, it can take off again only after replenishing energy and packages.
3. Each task point must be delivered to, can only be delivered to once by either a UAV or a truck, and cannot be delivered to repeatedly.
4. Each UAV flight can deliver to one or more task points. The total distance of each UAV flight task shall not exceed the maximum flight distance ($L_{max}$) of the UAV, and the total weight of the carried packages shall not exceed the total load ($M_{max}^{u}$) of the UAV.
5. The total weight of the packages carried by the truck for each task shall not exceed the total load ($M_{max}^{t}$) of the truck.

In the task environment, the packages at each task point may be delivered by UAVs or trucks. Referring to [23], the formulas for UAVs and trucks are defined as follows:

(1) $$\begin{array}{cc} x(a,b) & =\left\{\begin{array}{cc} 1, & \mathrm{The}\;\mathrm{UAV}\;\mathrm{will}\;\mathrm{fly}\;\mathrm{from}\;\mathrm{task}\;\mathrm{point}\;a\;\mathrm{to}\;\mathrm{task}\;\mathrm{point}\;b\\ 0, & \mathrm{The}\;\mathrm{UAV}\;\mathrm{will}\;\mathrm{not}\;\mathrm{fly}\;\mathrm{from}\;\mathrm{task}\;\mathrm{point}\;a\;\mathrm{to}\;\mathrm{task}\;\mathrm{point}\;b \end{array}\right. \end{array}$$

(2) $$\begin{array}{cc} y(a,b) & =\left\{\begin{array}{cc} 1, & \mathrm{The}\;\mathrm{truck}\;\mathrm{will}\;\mathrm{travel}\;\mathrm{from}\;\mathrm{task}\;\mathrm{point}\;a\;\mathrm{to}\;\mathrm{task}\;\mathrm{point}\;b\\ 0, & \mathrm{The}\;\mathrm{truck}\;\mathrm{will}\;\mathrm{not}\;\mathrm{travel}\;\mathrm{from}\;\mathrm{task}\;\mathrm{point}\;a\;\mathrm{to}\;\mathrm{task}\;\mathrm{point}\;b \end{array}\right. \end{array}$$

The constraints that need to be satisfied for the task-planning problem of the truck-independent UAV system are defined as follows:

1. Each task point must be delivered to and can only be delivered to once by either a UAV or a truck, without repetition, that is,
  (3) $$\sum_{a,b\in S}\left(x\left(a,b\right)+y\left(a,b\right)\right)=F,$$
2. Each UAV flight can deliver to one or more task points. The total distance of each UAV flight task shall not exceed the maximum flight distance ($L_{max}$) of the UAV, and the total weight of the carried packages shall not exceed the total load ($M_{max}^{u}$) of the UAV. We use $R_{U}=\left\{R_{U_{1}},R_{U_{2}},\ldots ,R_{U_{N}}\right\}$ to represent the set of task sets for *N* UAVs and $R_{U_{k}}=\left\{R_{U_{k1}},R_{U_{k2}},\ldots \right\}$ to represent the route arranged for UAV $U_{k}$, where $R_{U_{ki}}$ represents a flight task of the UAV. Therefore, for each flight task ($R_{U_{ki}}$) of the UAV, its total flight distance ($L_{R_{U_{ki}}}$) should be less than $L_{max}$, and its total load ($M_{R_{U_{ki}}}$) should be less than $M_{max}^{u}$, that is,
  (4) $$\begin{array}{cc} L_{R_{U_{ki}}} & <L_{max},\;\forall R_{U_{ki}}\in R_{U_{k}}, \end{array}$$
  (5) $$\begin{array}{cc} M_{R_{U_{ki}}} & <M_{max}^{u},\;\forall R_{U_{ki}}\in R_{U_{k}}, \end{array}$$
3. The total weight of the packages carried by the truck for each task shall not exceed the total load ($M_{max}^{t}$) of the truck. We use $R_{T}=\left\{R_{T_{1}},R_{T_{2}},\ldots ,R_{T_{E}}\right\}$ to represent the set of task sets for *E* trucks and $R_{T_{k}}=\left\{R_{T_{k1}},R_{T_{k2}},\ldots \right\}$ to represent the route arranged for truck *k*, where $R_{T_{kj}}$ represents the route of a single task of truck *k*. The following conditions need to be met:
  (6) $$M_{R_{T_{kj}}}<M_{max}^{t},\;\forall R_{T_{kj}}\in R_{T_{k}}.$$

Let dist(a,b) denote the Euclidean distance between task point *a* and task point *b*. Then, the flight distance ($L_{R_{U_{ki}}}$) of the flight task ($R_{U_{ki}}$) of UAV $U_{k}$ is given by

(7) $$L_{R_{U_{ki}}}=\sum_{a,b\in R_{U_{ki}}}x\left(a,b\right)dist\left(a,b\right),\;\forall R_{U_{ki}}\in R_{U_{k}}.$$

The travel distance of a single task ($R_{T_{kj}}$) of truck $T_{k}$ is:

(8) $$L_{R_{T_{kj}}}=\sum_{a,b\in R_{T_{kj}}}y\left(a,b\right)dist\left(a,b\right),\;\forall R_{T_{kj}}\in R_{T_{kj}}.$$

The objective of the task-planning problem for the truck-independent UAV system is to minimize the energy consumption of UAVs and trucks. According to the aircraft energy consumption model in aerodynamics theory, when a UAV flies at a fixed altitude, its propulsion energy consumption is a function of the flight trajectory. Referring to [18], we ignore the communication energy consumption of the UAV during flight and the change in the effective load during the delivery process. Therefore, the energy consumption of a single flight task ($R_{U_{ki}}$) of UAV $U_{k}$ can be expressed as follows:

(9) $$W_{R_{U_{ki}}}=\left(w_{d}+G\right)\frac{L_{R_{U_{ki}}}P}{370\eta \gamma \left(P-e\right)},\;\forall R_{U_{ki}}\in R_{U_{k}},$$

where $w_{d}$ represents the self-weight of the UAV, *G* represents the effective load, *P* represents the maximum power of the UAV, η represents the conversion efficiency of the UAV’s motor, γ represents the lift ratio, and *e* represents the energy loss of the UAV’s battery. Furthermore, the total energy consumption ($W_{U}$) of all UAVs can be expressed as follows:

(10) $$W_{U}=\sum_{R_{U_{k}}\in R_{U}}\sum_{R_{U_{ki}}\in R_{U_{k}}}W_{R_{U_{ki}}},\;i=1,2,\ldots ,\left|R_{U_{k}}\right|.$$

We define $\rho_{v}$ as the energy consumption per unit distance traveled by a truck. Thus, the total energy consumption of a truck can be obtained as follows:

(11) $$W_{T}=\sum_{R_{T_{k}}\in R_{T}}\sum_{R_{T_{kj}}\in R_{T_{k}}}\rho_{v}L_{R_{T_{kj}}},\;j=1,2,\ldots ,\left|R_{T_{k}}\right|.$$

Based on the above description of the task-planning problem for the truck-independent UAV system, as well as the definitions of the constraints and the objective function, it can be seen that our goal is to minimize the total energy consumption of the system under the constraints of the UAVs’ load and energy limitations and the truck’s load limitation. This problem not only involves a combinatorial optimization problem among various task points but also includes the shortest path problem, which is an optimization problem under multiple constraints. Through the above analysis, the task-planning problem for the truck-independent UAV system can be described as follows:

(12) $$\begin{array}{cc} \; & \min W=W_{U}+W_{T}\\ \; & s.t.\;(3)–(6) \end{array}$$

## 4. Proposed Algorithm

In the previous section, we analyzed the logistics task allocation problem of a truck-independent UAV system in a known environment and established a mathematical model. In this section, we mainly design corresponding solution methods to solve the above problem model.

### 4.1. Sub-Path Allocation

This section discusses how to assign the task points in the scenario to UAVs and trucks under the constraints of load and energy and reasonably arrange each of their tasks so as to minimize the total energy consumption of the system while completing all tasks. Our problem is similar to the multi-task problem with few agents proposed in [25], which involves not only the combinatorial optimization problem of depots and task points but also the shortest path problem. Differing from the multi-task problem with few agents, we require that each task point be delivered to—and only once. Nevertheless, we can still obtain all possible combinations of depots and task points in advance and obtain a better solution to the combinatorial optimization problem by constructing the minimum-cost, maximum-flow (MCMF) model [26,27].

Before transforming the task-planning problem into the MCMF problem, it is necessary to first define each task of UAVs and trucks. Each time UAVs and trucks execute a task, they need to take off from a depot, complete the delivery tasks, and land at a depot. We define the depot–task set–depot combination as $LSL=\{D_{1},D_{2},r_{1},r_{2},\ldots \}$, where the first two elements ($D_{1}$ and $D_{2}$) are depots and the third and subsequent elements represent the delivery tasks to be executed in one task, with a variable number of delivery tasks each time.

The minimum-cost, maximum-flow problem aims to find an optimal path with the minimum cost and the maximum flow. Therefore, we choose to transform the task-planning problem into the minimum-cost, maximum-flow problem, that is, to find the LSL combination with the minimum energy consumption that covers all task points. We construct a new MCMF model according to the constraints in the problem and use the minimum-cost, maximum-flow theory to obtain the optimal LSL combination. However, the ordinary MCMF model cannot be directly used, and several issues still need attention [28]:

- It is impossible to solve the problem with only task points in the flow network?
- How should the path of each UAV flight task be represented in the model?
- How should the requirements of UAV load and energy be embodied in the model?

**Figure 2:**
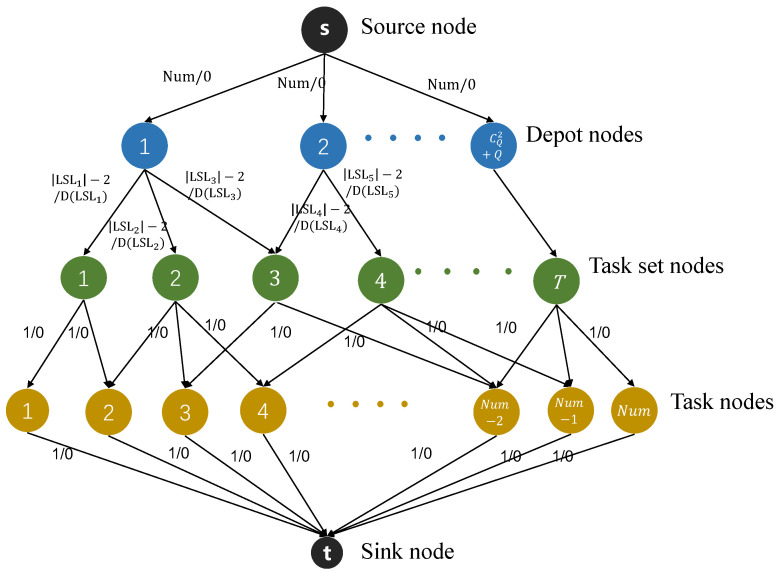
The MCMF model for the task-planning problem of the truck-independent UAV system.

Considering the three problems mentioned above, we can construct MCMF models for UAVs and trucks. The construction methods for are the same for both. We take a UAV as an example, as shown in Figure 2, denoted as $G_{U}$. It is known that the flow network of the MCMF model is a flow–cost network graph, where each edge has two attributes: capacity and cost. The capacity of an edge represents the maximum flow that can pass through this edge, and the cost represents the cost per unit of flow passing through this edge. We model the number of completed tasks as the flow and use the flight distance of a single UAV flight task to represent the cost on the corresponding edge. Next is a detailed description of the flow–cost network graph ($G_{U}$).

The edges connecting each node have two attributes: capacity and cost. We set different capacities and costs for each edge to meet the load and energy limitations of the UAV. The nodes in graph $G_{U}$ can be classified into four categories. One category consists of the auxiliary nodes added to the model, which serve as the source and sink nodes of graph $G_{U}$. The other three categories of nodes are depot combination nodes, task-set nodes, and task nodes. Detailed descriptions of these three categories of nodes are presented as follows:

1. Depot combination nodes: Since the UAVs are independent, considering the energy and load limitations, a UAV needs to land at a nearby depot to recharge and restock packages before its energy is exhausted. The take-off depot and the landing depot can be either the same or different. For trucks, due to their load limitations, they can only carry a certain weight of packages each time. After finishing a delivery task, if they need to start another trip, they also have to reach a depot to replenish packages. Similarly, the starting depot and the ending depot of a truck can be the same or different. Consequently, each depot combination contains the starting and ending points of a single delivery task for either a truck or a UAV. These starting and ending points may be identical or distinct. In total, there are $C_{Q}^{2}+Q$ depot combination nodes. As shown in Figure 2, we add these depot combinations to the graph ($G_{U}$) as first-level nodes, namely depot combination nodes, which are denoted by blue dots. Therefore, there are $C_{Q}^{2}+Q$ depot combination nodes in graph $G_{U}$. We define the set of depot combinations as $DD=\{DD_{1},DD_{2},\ldots ,DD_{C_{Q}^{2}+Q}\}$, where $DD_{k}=\left\{D_{i},D_{j}\right\}$ with i,j∈{1,2,…,Q} representing a depot combination.
2. Task-set nodes: Due to the limitations of the UAV’s load and energy, the number of tasks that a UAV can execute in each flight is limited. Therefore, the tasks assigned to the UAV in the scenario need to be carried out in multiple flights. For a UAV, different depot combinations can result in different LSLs for the task points it is responsible for, and these LSLs are all possible flight tasks for the UAV. Our goal is to select several LSLs among these candidates. These selected LSLs should cover all the tasks assigned to the UAV in the scenario while minimizing the UAV’s energy consumption, and there should be no overlap in the task points among different LSLs. The task points assigned to the UAV and all the depots in the scenario can form a complete undirected graph, where the weight of each edge is the Euclidean distance between two task points. For all depot combinations, the depth-first search algorithm is used to obtain all paths between any two depots in the complete undirected graph. Considering the load and energy limitations of the UAV, all paths with a total distance greater than $L_{max}$ and a total load greater than $M_{max}^{u}$ are removed. Thus, the remaining paths are all feasible paths for the UAV. Each path can be converted into an LSL combination, and all the task points in the combination can form a task set. We list the task sets of all feasible paths and add them to graph $G_{U}$ as second-level nodes, that is, task-set nodes, which are represented by green dots, as shown in Figure 2. Assuming that the total number of feasible paths for the UAV in the scenario is *T*, there are *T* task-set nodes in graph $G_{U}$. The same principle applies to trucks.
3. Task nodes: Since the tasks contained in each task set cannot be reflected in the nodes, we add all the task points that the UAV is responsible for to the graph ($G_{U}$) as task nodes, which are third-level nodes and represented by yellow dots, as shown in Figure 2. Each task node contains only one task point. Taking the UAV as an example, assuming that the number of task points that the UAV is responsible for is Num, there are Num task nodes in graph $G_{U}$. The same applies to trucks.

In addition, we use different capacities and costs to represent the different requirements of each edge. Introductions to the various types of edges are presented as follows:

1. Edges between depot combination nodes and task-set nodes: As mentioned previously, the depth-first search algorithm can be used to obtain all feasible paths of the UAV and the total length of each feasible path. Each feasible path is composed of a task-set node and a depot combination node. If a task-set node and a specific depot combination node can form a feasible path, there exists an edge between this task-set node and this depot combination node. The cost of this edge represents the total length of the corresponding feasible path. For an LSL combination ($LSL_{k}$), the corresponding set of task points may have different execution orders. We select the execution order with the minimum total distance as the final result and record this distance as $D(LSL_{k})$. In this paper, a depot combination may plan multiple UAV routes simultaneously, that is, an edge may exist between a depot combination node and multiple task-set nodes. Aside from these edges, the cost of edges between other nodes is 0 because the other edges represent the corresponding relationships between different nodes rather than the moving distance. In addition, the capacity of the edge between a depot combination node and a task-set node is the number of task points in the task-set node, that is, $|LSL_{k}|-2$. It should be noted that the flow on the edge between a depot combination node and a task-set node is either 0 or $|LSL_{k}|-2$, and the flow cannot be adjusted gradually because each UAV flight task is completed at one time.
2. Edges between the source node and depot combination nodes: As we know from the previous content, each depot combination node may be connected to multiple task-set nodes. Therefore, it is possible that the starting point and the ending point of each task are the same depot combination. Taking a UAV as an example, assuming that the number of task points that the UAV is responsible for is Num, the maximum capacity of the edge between the source node and the depot combination node in the minimum-cost, maximum-flow model of the UAV is the total number of task points Num, as shown in Figure 2.
3. Edges between task-set nodes and task nodes: The edges between task-set nodes and task nodes represent the corresponding relationship between task sets and tasks, that is, a task-set node is only connected to the task nodes corresponding to the task points it contains. Therefore, the capacity of the edge between a task-set node and a task node is 1, and the cost is 0.
4. Edges between task nodes and the sink node: The capacity of the edge between a task node and the sink node is set to 1, which means that each task point can only be executed once. Similarly, the cost of the edge between them is 0.

In summary, based on the assumptions made above, we can construct a flow network ($G_{U}=\left(V,E,C,\omega \right)$, where *C* represents the capacity of each edge and ω represents the total cost of each edge). We propose an algorithm for selecting the LSL combination with the shortest total path from the MCMF model. The pseudocode of the algorithm is shown in Algorithm 1.

First, as shown in lines 1–7 of Algorithm 1, we need to construct the capacity network (G=(V,E,C,ω)) of the MCMF model according to the task points in the scenario and all depots and initialize the flow (*f*) as the path corresponding to the LSL with the minimum cost. We calculate the total cost of the final feasible flow obtained by continuously searching for augmenting paths in the remaining network without adjustment, with *f* as the initial flow. This total cost is $cost_{min}$. Thus, we can obtain the total cost of the final flow obtained with *f* as the initial flow in $G_{f}$.
**Algorithm 1** Allocate depot–task set–depot combinations for UAVs**Input**: Depot combinations *DD*, the set of task points $S_{U}$ that the UAV is responsible for.**Output**: The set of *LSL*s contained in *f*, denoted as $LSL_{u}=\left\{LSL_{u}^{1},LSL_{u}^{2},\ldots \right\}$1. Initialize the capacity network $G_{U}=\left(V,E,C,\omega \right)$ of the UAV’s MCMF model.2. **Function** $Cost_{f}$ (the feasible flow *f*)3.       **While** there are still augmenting paths in the capacity network $G_{f}$4.             Select an augmenting path $p^{*}$ that does not conflict with *f* from the incremental network $G_{f}^{\prime }$ of *f*;5.             Increase the flow of *f* according to $p^{*}$.6.       **End While**7. **Return** *f*, the total cost of *f*.8. Initialize *f* as the path corresponding to the LSL with the minimum cost, and the capacity is the number of task points in this LSL;9. $cost_{min}=Cost_{f}\left(f\right)$;10. **For** take out $LSL_{k}$ in ascending order of cost11.       **If** the task points in $LSL_{k}$ do not conflict with *f*12.             Increase the flow of *f* according to the path where $LSL_{k}$ is located;13.       **Else**14.             $f_{LSL_{k}}=f$;15.             Release all paths where the task points that conflict with $LSL_{k}$ are located in $f_{LSL_{k}}$, and increase the flow of $f_{LSL_{k}}$ according to $LSL_{k}$;16.             $\left(f_{LSL_{k}},cost_{LSL_{k}}\right)=Cost_{f}\left(f_{LSL_{k}}\right)$;17.             **If** $cost_{LSL_{k}}<cost_{min}$18.                   Delete all paths from *s* to *t* in $f_{LSL_{k}}$ whose costs are greater than those of $LSL_{k}$, and let $f=f_{LSL_{k}}$.19.             **End If**20.       **End If**21. **End For**

Afterwards, as shown in lines 8–21 of Algorithm 1, we take out the combinations ($LSL_{k}$) of depot combination nodes and task-set nodes in graph *G* in ascending order of the total cost. If the task points in $LSL_{k}$ do not conflict with *f*, then the flow of *f* is increased according to $LSL_{k}$; otherwise, it is necessary to judge whether *f* needs to be adjusted. Let $f_{LSL_{k}}=f$; then, all paths where the task points that conflict with $LSL_{k}$ are located in $f_{LSL_{k}}$ are released, and the flow of $f_{LSL_{k}}$ is increased according to the path where $LSL_{k}$ is located. The final feasible flow ($f_{LSL_{k}}$) is obtained by continuously searching for augmenting paths without adjustment, with $f_{LSL_{k}}$ as the initial flow and a total cost of $Cost_{LSL_{k}}$. If $Cost_{LSL_{k}}<cost_{min}$, there is a better initial flow than the result obtained by using *f* as the initial flow. All paths corresponding to the RSR in $f_{LSL_{k}}$ whose costs are greater than those of $LSL_{k}$ are deleted, and the adjusted $f_{LSL_{k}}$ is taken as the new initial flow, that is, $f=f_{LSL_{k}}$. Eventually, the optimal solution can be found in the capacity network (*G*).

In Algorithm 1, according to the backtracking idea in the MCMF theory, the combinations of the LSL values are continuously adjusted until the set of LSLs with the minimum total cost, denoted as $LSL_{u}$, is obtained. The set of depot–task set–depot combinations that cover all the task points of UAVs and have the minimum cost in the scenario is obtained and denoted as $LSL_{u}=\left\{LSL_{u}^{1},LSL_{u}^{2},\ldots \right\}$, where $LSL_{u}^{k}$ represents the *k*-th feasible path of the UAV. Similarly, for trucks, the set of depot–task set–depot combinations that cover all the task points of trucks and have the minimum cost can be obtained and denoted as $LSL_{t}=\left\{LSL_{t}^{1},LSL_{t}^{2},\ldots \right\}$, where $LSL_{t}^{k}$ represents the *k*-th feasible path of the truck. It can be concluded that the time complexity of Algorithm 1 is $O\left(Q^{2}Num^{2}\right)+O\left(TQNum^{2}\left(Num+Q+T\right)\right)$. The term $O(Q^{2}Num^{2})$ represents the complexity of finding all feasible LSLs through the depth-first search algorithm. The term $O(TQNum^{2}\left(Num+Q+T\right))$ represents the complexity of finding the optimal solution in the flow network (*G*) of the MCMF model.

### 4.2. Initial Route Planning

As mentioned above, we construct the MCMF model for the task-planning problem of the truck-independent UAV system to obtain a set of all feasible paths. This set includes the feasible paths ($LSL_{t}$) for trucks and the feasible paths ($LSL_{u}$) for UAVs. Since the problem of planning paths for UAVs or trucks according to the feasible paths is the same, the following takes path planning for UAVs as an example to describe the algorithm in detail. There are *N* UAVs in the scenario. Therefore, we need to construct all the LSLs in $LSL_{u}$ as continuous paths whose number is no more than *N*. The same principle applies to trucks.

**Figure 3:**
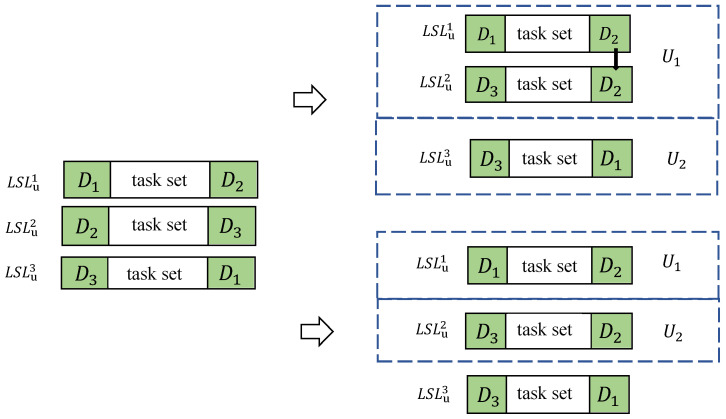
Constructing multi-UAV paths using the matching method.

Before planning paths for UAVs, it should be noted that the first flight mission, i.e., the first depot–task set–depot combination, selected by each UAV departing from its initial depot often has an impact on subsequent path selections. As shown in Figure 3, assume that the initial docking depots of UAVs $U_{1}$ and $U_{2}$ in the scenario are $D_{1}$ and $D_{3}$, respectively. If $U_{1}$ chooses to execute $LSL_{u}^{1}$ first and $U_{2}$ chooses to execute $LSL_{u}^{3}$ first, then $U_{1}$ will land at $D_{2}$ and can continue to execute $LSL_{u}^{2}$. If $U_{1}$ chooses to execute $LSL_{u}^{1}$ first and $U_{2}$ chooses to execute $LSL_{u}^{2}$ first, then both $U_{1}$ and $U_{2}$ will land at $D_{2}$. At this time, since the depot combination of $LSL_{u}^{3}$ does not contain $D_{2}$, neither $U_{1}$ nor $U_{2}$ can continue to execute $LSL_{u}^{2}$. Therefore, there is a possibility that these combinations cannot form continuous executable paths containing the maximum number of LSLs simply by the matching method.

To solve this problem, for all the LSLs obtained previously, according to the initial docking depots of each UAV, we propose an algorithm using a resource tree [29] to obtain all the sequences of LSLs that can be executed sequentially, that is, the initial paths of *N* UAVs. Then, the insertion method is used to find the optimal insertion points for the feasible paths that are not included in the initial paths. When obtaining the sequences of LSLs that can be executed sequentially as the initial paths for all UAVs, the main consideration is whether the depots in every two LSLs overlap. The overlapping depots can serve as the landing depot of the previous LSL and the take-off depot of the subsequent LSL, while the order of the task sets does not need to be considered. Therefore, we simplify each LSL to the corresponding depot combination ($DD_{k}$), and depot combinations with different depot orders can be regarded as the same depot combination. It should be noted that different LSLs may have the same depot combination. Therefore, a parameter ($Num_{k}$) is set for each depot combination ($DD_{k}$) to represent the number of the depot combinations in $LSL_{u}$, which is used to judge whether the depot combination can be added to the path.

First, according to the initial docking depots of each UAV, we can obtain all depot combinations that contain the initial docking depots. By performing a full permutation of the depot combinations for each UAV, we can determine all the possibilities of the first flight mission sets for each UAV. Afterwards, according to the $Num_{k}$ parameter, the sets of depot combinations that exceed the upper limit are deleted. Taking Figure 3 as an example, the set of depot combinations is $DD=\{DD_{1},DD_{2},DD_{3}\}$, where $DD_{1}=\left\{D_{1},D_{2}\right\}$, $DD_{2}=\left\{D_{2},D_{3}\right\}$, and $DD_{3}=\left\{D_{3},D_{1}\right\}$. Their corresponding parameters are $Num_{1}=1$, $Num_{2}=1$, and $Num_{3}=1$, respectively. Assume that the initial docking depots of UAV $U_{1}$ and $U_{2}$ are $D_{1}$ and $D_{3}$, respectively. Then, the paths that can be the first flight mission of $U_{1}$ are $LSL_{u}^{1}$ and $LSL_{u}^{3}$, and the paths that can be the first flight mission of $U_{2}$ are $LSL_{u}^{2}$ and $LSL_{u}^{3}$. Therefore, all the combinations of the first flight missions of UAV $U_{1}$ and $U_{2}$ are $\left\{LSL_{u}^{1},LSL_{u}^{2}\right\}$, $\left\{LSL_{u}^{1},LSL_{u}^{3}\right\}$, $\left\{LSL_{u}^{3},LSL_{u}^{2}\right\}$, and $\left\{LSL_{u}^{3},LSL_{u}^{3}\right\}$. Their corresponding depot combinations are $\left\{\left\{D_{1},D_{2}\right\},\left\{D_{3},D_{2}\right\}\right\}$, $\left\{\left\{D_{1},D_{2}\right\},\left\{D_{3},D_{1}\right\}\right\}$, $\left\{\left\{D_{1},D_{3}\right\},\left\{D_{3},D_{2}\right\}\right\}$, and $\left\{\left\{D_{1},D_{3}\right\},\left\{D_{3},D_{1}\right\}\right\}$, respectively. Obviously, in $\left\{\left\{D_{1},D_{3}\right\},\left\{D_{3},D_{1}\right\}\right\}$, there is a repeated depot combination ($\left\{D_{1},D_{3}\right\}$), and it is known that $Num_{3}=1$, which means that there is only one depot combination ($\left\{D_{1},D_{3}\right\}$) in $LSL_{u}$, and it can only be used once. Therefore, $\left\{\left\{D_{1},D_{3}\right\},\left\{D_{3},D_{1}\right\}\right\}$, which uses the combination twice, does not meet the requirements and is removed.

After obtaining all the possibilities of the first depot combination sets (flight mission sets) for each UAV, these sets are used as the first-level nodes of the resource tree, and an auxiliary node *s* is used as the root node of the tree to connect all the first-level nodes, thereby forming the initial state of the resource tree. Let the initial path be Path=Φ. The algorithm based on the resource tree combined with the depth-first search idea is used to select the initial path for the UAV. The pseudocode of the algorithm is shown in Algorithm 2, and the main steps are outlined as follows:

1. Initialize Stack as an empty stack, and take out the root node (*s*) and its first direct child node and put them into Stack. Except for the root node, the nodes stored in Stack are all sets of depot combinations.
2. Take out the first node from Stack as the current node, obtain the set of its depot combinations, and successively subtract 1 from the parameter ($Num_{k}$) corresponding to each depot combination ($DD_{k}=\left\{D_{i},D_{j}\right\}$) in the set, indicating that the usage frequency of this combination is reduced by one. After that, check whether this set can be used to assign the next flight mission for the UAV, that is, to add the next-level node. The inspection criteria are as described follows: If all the parameters corresponding to the combinations in DD are 0, all depot combinations have been assigned, that is, the path from the root node to the current node is the appropriate initial path, denoted as *Path* = Stack excluding the first root node + the set of depot combinations of the current node, and the algorithm ends; otherwise, for this set, execute step 3.
3. For each depot combination ($DD_{k}=\left\{D_{i},D_{j}\right\}$) in the set of depot combinations, search in DD for depot combinations that contain $D_{j}$. Depot combinations with a parameter equal to 0 are not considered. Let $DD_{k}^{temp}=\Phi$ be used to store all depot combinations that may serve as the next flight mission for $DD_{k}$. If there is one or more combinations that meet the requirements, execute step 4; if there are no such combinations, do not operate on $DD_{k}^{temp}$. After traversing all combinations, execute step 5.
4. Judge each combination ($DD_{\eta }=\left\{D_{j},D_{l}\right\}$) in $DD_{k}^{temp}$ in turn. If $Num_{\eta }>0$, $DD_{\eta }$ can be used as the next flight mission. Let $DD_{k}^{temp}=DD_{k}^{temp}+\left\{DD_{\eta }\right\}$. After the traversal is completed, return to step 3.
5. If $DD_{k}^{temp}$ for each combination is NULL, this node cannot find the next-level node downward. Then, it is necessary to judge whether the sum of the parameters corresponding to all combinations in the current DD is smaller than the sum of the parameters corresponding to Path. If it is smaller than the sum of the parameters corresponding to Path, the current path contains more depot combinations and is a better initial path. Let *Path* = Stack excluding the root node + the set of depot combinations corresponding to the current node, and execute step 7; otherwise, perform a full permutation on $DD_{k}^{temp}$ corresponding to each $DD_{k}$ to obtain sets of multiple depot combinations. If $DD_{k}^{temp}$ is empty, fill it with Null during the permutation, then execute step 6.
6. Obtain a set in turn, and make the following judgments for each depot combination ($DD_{\lambda }$) in the set: If the $Num_{\lambda }>0$, subtract 1 from $Num_{\lambda }$. At this time, if all combinations in the set have been traversed, add this set as a child node of the current node to the resource tree and mark it as unvisited; If the $Num_{\lambda }$ parameter of $DD_{\lambda }$ is 0, this depot combination cannot be used anymore, that is, $DD_{\lambda }$ is infeasible. Directly judge the next set. After the traversal of all sets is completed, execute step 7.
7. If there are unvisited direct child nodes of the current node, add the current node and these direct child nodes to Stack in turn, and execute step 2; otherwise, judge whether there are unvisited direct child nodes of the parent node of the current node. If there is still none, continue to search upward according to step (7), and at the same time, remove the corresponding nodes from Stack. During this process, continuously reverse all parameter adjustments until Stack is empty, and the algorithm ends.

**Figure 4:**
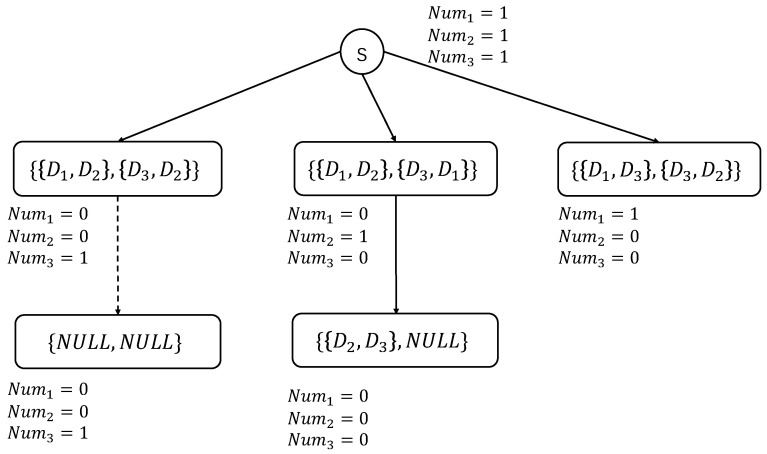
Finding the initial route of the UAV based on the resource tree.

To better understand Algorithm 2, we give provide a simple example for illustration. The constructed resource tree is shown in Figure 4. Still taking Figure 3 as an example, assume that the initial docking depots of UAVs $U_{1}$ and $U_{2}$ are $D_{1}$ and $D_{3}$, respectively. As mentioned above, the sets of all depot combinations for the first flight mission of each UAV are $\left\{\left\{D_{1},D_{2}\right\},\left\{D_{3},D_{2}\right\}\right\}$, $\left\{\left\{D_{1},D_{2}\right\},\left\{D_{3},D_{1}\right\}\right\}$, and $\left\{\left\{D_{1},D_{3}\right\},\left\{D_{3},D_{2}\right\}\right\}$. In the initially constructed resource tree, there is a root node (*s*) and three child nodes of the root node, which are the above three sets of depot combinations. Therefore, we initialize $Stack=\{\left\{\left\{D_{1},D_{2}\right\},\left\{D_{3},D_{2}\right\}\right\},s\}$ and Path=Φ. At this time, the sum of the parameters corresponding to Path is 3. Then, we the first node ($\left\{\left\{D_{1},D_{2}\right\},\left\{D_{3},D_{2}\right\}\right\}$) from Stack as the current node. After subtracting 1 from the parameters corresponding to the depot combinations in the current node to indicate that they have been used once, Stack={s}. At this time, there are two depot combinations in the current node, and we judge them one by one. First, we perform the corresponding operation on $\left\{D_{1},D_{2}\right\}$, let $Num_{1}=0$, and search for depot combinations that contain $D_{2}$ among the remaining depot combinations. Since the parameters of both $\left\{D_{1},D_{2}\right\}$ and $\left\{D_{2},D_{3}\right\}$ are 0 at this time, we obtain $DD_{1}^{temp}=\Phi$. Similarly, $DD_{2}^{temp}=\Phi$. Both $DD_{1}^{temp}$ and $DD_{2}^{temp}$ being empty means that this node cannot find the next-level node downward. It is easy to know that the sum of the parameters is 1 at this time and is less than 3, so we record $Path=\{\left\{\left\{D_{1},D_{2}\right\},\left\{D_{3},D_{2}\right\}\right\}\}$, and the sum of the parameters corresponding to Path is 1 at this time. Since there are no unvisited child nodes of the current node but its parent node has an unvisited direct child node $\left\{\left\{D_{1},D_{2}\right\},\left\{D_{3},D_{1}\right\}\right\}$, $Stack=\{\left\{\left\{D_{1},D_{2}\right\},\left\{D_{3},D_{1}\right\}\right\},s\}$, and the parameter adjustments of the state of the parent node of this node are reversed, that is, $Num_{1}=1$, $Num_{2}=1$, and $Num_{3}=1$.
**Algorithm 2** Construct the Initial Path**Input**: The set of depot combinations $DD=\{DD_{1},DD_{2},\ldots ,DD_{N}\}$ composed of $LSL_{u}$ or $LSL_{t}$.**Output**: The initial path Path.1. Initialize stack *Stack* = {the first direct child node of *s*, *s*}, Path=Φ.2. **While** Stack≠Φ3.       Take the first node from Stack as the current node.4.       **For** each depot combination $DD_{k}=\left\{D_{i},D_{j}\right\}$ in the set of depot combinations5.             $Num_{k}=Num_{k}-1$, $DD_{k}^{temp}=\Phi$.6.             **If** it is the final result of assigning all depot combinations7.                   Record the result with Path and end the algorithm.8.             **Else**9.                   Select the candidate depots for constructing the next node for each UAV,10.                  Record them with $DD_{k}^{temp}$.11.             **End If**12.             **If** $DD_{k}^{temp}$ for each combination is empty13.                   **If** the sum of the parameters in the current DD < Path14.                         Let Path=Stack excluding the first root node + the set of depot combinations of the current node.15.                   **End If**16.             **Else**17.                   Perform a full permutation on $DD_{k}^{temp}$ corresponding to each $DD_{k}$.18.                   **If** the obtained sets of depot combinations can be used as child nodes of the current node19.                       Add them to the resource tree and set their status to unvisited.20.                   **End If**21.             **End If**22.       **End For**23.       **If** there are unvisited direct child nodes of the current node24.             Add the current node and these direct child nodes to Stack in turn.25.       **Else**26.             keep looking upward for the parent node with unvisited direct child nodes,27.             Remove the corresponding nodes from Stack and reverse the status of all parameters until Stack=Φ.28. **End While**

If Stack is not empty, the first node ($\left\{\left\{D_{1},D_{2}\right\},\left\{D_{3},D_{1}\right\}\right\}$) from Stack is taken as the current node again. After performing corresponding operations on the parameters, Stack={s}. There are two depot combinations in the current node, and we judge them one by one. First, we perform the corresponding operation on $\left\{D_{1},D_{2}\right\}$, let $Num_{1}=0$, and search for depot combinations that contain $D_{2}$ in DD. Since the $Num_{1}$ parameter of $\left\{D_{1},D_{2}\right\}$ is 0, it cannot be added to $DD_{1}^{temp}$, and the $Num_{2}$ parameter of $\left\{D_{2},D_{3}\right\}$ is 1, so it can be added to $DD_{1}^{temp}$, that is, $DD_{1}^{temp}=\left\{\left\{D_{2},D_{3}\right\}\right\}$. Similarly, $DD_{3}^{temp}=\Phi$. Since $DD_{1}^{temp}$ and $DD_{3}^{temp}$ are not both empty, the result of the full permutation of them to obtain sets of depot combinations is $\left\{\left\{\left\{D_{2},D_{3}\right\},Null\right\}\right\}$. Next, we need to judge each obtained set of depot combinations to see whether it can become a child node of the tree. For $\left\{\left\{D_{2},D_{3}\right\},Null\right\}$, Null has no parameter, and we change the parameter of $\left\{D_{3},D_{2}\right\}$ to $Num_{2}=0$. At this time, the parameters of all combinations in the set are greater than or equal to 0. Therefore, this set can be inserted into the resource tree as a child node of the current node, and the status of this child node is set to unvisited. Since there is an unvisited child node ($\left\{\left\{D_{2},D3\right\},Null\right\}$) of the current node, we add the current node and this direct child node to Stack in turn. Then, $Stack=\{\left\{\left\{D_{2},D3\right\},Null\right\},\left\{\left\{D_{1},D2\right\},\left\{D_{3},D1\right\}\right\},s\}$.

We take the first node ($\left\{\left\{D_{2},D3\right\},Null\right\}$) from Stack as the current node again. It is easy to know that the sum of all adjusted parameters is 0, which means that all depot combinations in DD have been assigned. Therefore, the path from the root node to the current node, excluding the root node, is the final initial path, and we do not continue to look for other possibilities. Therefore, the initial path is $Path=\{\left\{\left\{D_{1},D2\right\},\left\{D_{3},D1\right\}\right\},\left\{\left\{D_{2},D3\right\},Null\right\}\}$. Each set of depot combinations represents a flight mission of a group of UAVs, that is, the initial path of UAV $U_{1}$ can be represented by depot combinations as $\left\{\left\{D_{1},D2\right\},\left\{D_{2},D3\right\}\right\}$, and the initial path of UAV $U_{2}$ can be represented by depot combinations as $\left\{\left\{D_{3},D1\right\}\right\}$.

After obtaining the initial paths of each UAV, we select the corresponding LSLs from $LSL_{u}$ according to the depot combinations to form a complete flight path. Therefore, the initial path of UAV $U_{k}$ can be defined as $R_{U_{k}}=\left\{LSL_{u}^{k1},LSL_{u}^{k2},\ldots \right\}$.

### 4.3. Global Route Adjustment

Considering that there may still be a small number of depot–task set–depot combinations that are not included after constructing the initial paths, it is necessary to insert the remaining depot–task set–depot combinations into the known initial paths. We refer to the remaining depot–task set–depot combinations as candidate depot–task set–depot combinations, denoted by the set expressed as $CLSL=\{LSL_{u}^{c1},LSL_{u}^{c2},\ldots \}$. In this section, we use the insertion method to select the optimal insertion point for each candidate combination in the initial paths of all UAVs in turn and insert the candidate depot–task set–depot combination into this optimal insertion point. The selection criterion for the optimal insertion point is that the total distance of the new path is the shortest after inserting the candidate combination.

**Figure 5:**
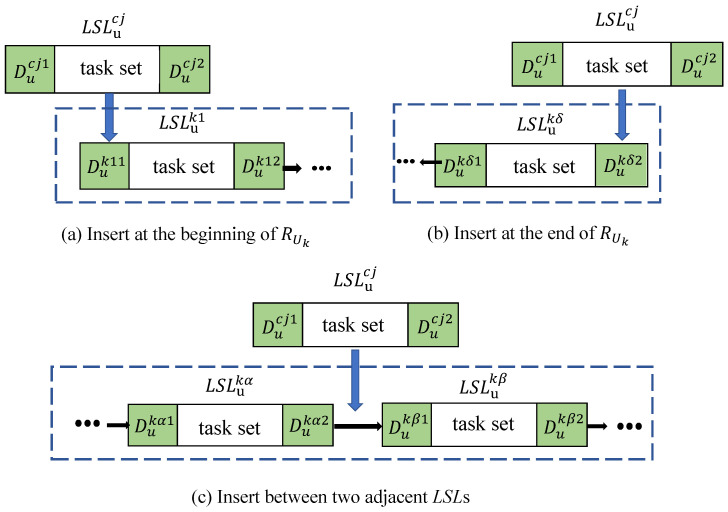
Three insertion point positions.

As shown in Figure 5, the candidate LSL can be inserted at the beginning or end of $R_{U_{k}}$ and between two adjacent LSLs. We list three different situations that exist when inserting the candidate LSL into the initial paths of each UAV and provide corresponding adjustment methods. The initial path ($R_{U_{k}}=\left\{LSL_{u}^{k1},LSL_{u}^{k2},\ldots ,LSL_{u}^{k\delta }\right\}$) of UAV $U_{k}$ contains $|R_{U_{k}}|$ depot–task set–depot combinations, which means there are $|R_{U_{k}}|+1$ insertion points. Taking the initial path ($R_{U_{k}}$) of UAV $U_{k}$ and the candidate depot—task set–depot combination ($LSL_{u}^{cj}$) as an example, the specific description is presented as follows:

1. Insertion at the beginning of $R_{U_{k}}$: The insertion position (*p*) is shown in Figure 5a. We use $D_{z}$ to denote the first take-off depot in $R_{U_{k}}$, that is, $D_{z}=D_{u}^{k11}$. If $D_{u}^{cj1}=D_{u}^{cj2}=D_{z}$, then the candidate depot–task set–depot combination ($LSL_{u}^{cj1}$) must already be in the initial path, so this situation will not occur.
  If $D_{u}^{cj1}=D_{z}$ and $D_{u}^{cj1}\neq D_{u}^{cj2}$, then we insert $LSL_{u}^{cj}$ directly into position *p* with $D_{u}^{cj1}$ as the take-off depot. Obviously, the distance from $D_{u}^{cj2}$ directly to the first task point in combination $LSL_{u}^{k1}$ is shorter than the distance from $D_{u}^{cj2}$ to $D_{z}$, then to the first task point in combination $LSL_{u}^{k1}$. Therefore, we choose to let the UAV take off from $D_{u}^{cj2}$, then directly execute the first task point in $LSL_{u}^{k1}$ instead of going to take-off point $D_{z}$. We stipulate that for a depot–task set–depot combination (*A*), when one of its depots (*a*) is replaced by another depot (*b*), the flight distance of the new *A* can be defined as $L^{b}\left(A,a\right)$. Thus, the total flight distance of the new combination after replacing depot $D_{z}$ in $LSL_{u}^{k1}$ with $D_{u}^{cj2}$ is $L^{D_{u}^{cj2}}\left(LSL_{u}^{k1},D_{z}\right)$. Therefore, after inserting the candidate combination ($LSL_{u}^{cj}$) into the beginning of $R_{U_{k}}$, the total distance of the new $R_{U_{k}}$ can be expressed as follows:
  (13) $$L\left(R_{U_{k}}\vee_{p}LSL_{u}^{cj}\right)\;=\;L\left(R_{U_{k}}\right)+L\left(LSL_{u}^{cj}\right)-L\left(LSL_{u}^{k1}\right)+L^{D_{u}^{cj2}}\left(LSL_{u}^{k1},D_{z}\right)$$
  where $L(R_{U_{k}}\vee_{p}LSL_{u}^{cj})$ represents the total length of the new $R_{U_{k}}$ after inserting combination $LSL_{u}^{cj}$ into position *p* of $R_{U_{k}}$, and $L(R_{U_{k}})$ and $L(LSL_{u}^{k1})$ represent the total distances of $R_{U_{k}}$ and $LSL_{u}^{k1}$, respectively. When $D_{u}^{cj2}=D_{z}$ and $D_{u}^{cj1}\neq D_{u}^{cj2}$, the same principle applies. We insert $LSL_{u}^{cj}$ directly into position *p* with $D_{u}^{cj2}$ as the take-off depot and, at the same time, replace the take-off depot of $LSL_{u}^{k1}$ with $D_{u}^{cj1}$.
  If $D_{u}^{cj1}\neq D_{z}$ and $D_{u}^{cj2}\neq D_{z}$, where $D_{u}^{cj1}$ and $D_{u}^{cj2}$ may be the same or different, it is necessary to calculate the total distances of the new $R_{U_{k}}$ after inserting $LSL_{u}^{cj}$ into position *p* when replacing $D_{u}^{cj1}$ with $D_{z}$ and replacing $D_{u}^{cj2}$ with $D_{z}$, then select the minimum one as the final result. At the same time, the take-off depot of $LSL_{u}^{k1}$ should be replaced with the depot in $LSL_{u}^{cj}$ that has not been replaced. The total flight distance of $R_{U_{k}}$ after inserting $LSL_{u}^{cj}$ is expressed as follows:
  (14) $$L^{D_{u}^{cj1}}\left(R_{U_{k}}\vee_{p}LSL_{u}^{cj}\right)\;=\;L\left(R_{U_{k}}\right)-L\left(LSL_{u}^{k1}\right)+L^{D_{u}^{cj2}}\left(LSL_{u}^{k1},D_{z}\right)+L^{D_{z}}\left(LSL_{u}^{cj},D_{u}^{cj1}\right),$$
  (15) $$L^{D_{u}^{cj2}}\left(R_{U_{k}}\vee_{p}LSL_{u}^{cj}\right)\;=\;L\left(R_{U_{k}}\right)-L\left(LSL_{u}^{k1}\right)+L^{D_{u}^{cj1}}\left(LSL_{u}^{k1},D_{z}\right)+L^{D_{z}}\left(LSL_{u}^{cj},D_{u}^{cj2}\right),$$
  (16) $$L\left(R_{U_{k}}\vee LSL_{u}^{cj}\right)\;=\;min\left\{L^{D_{u}^{cj1}}\left(R_{U_{k}}\vee_{p}LSL_{u}^{cj}\right),L^{D_{u}^{cj2}}\left(R_{U_{k}}\vee_{p}LSL_{u}^{cj}\right)\right\}.$$
  where $L^{D_{u}^{cj1}}\left(R_{U_{k}}\vee_{p}LSL_{u}^{cj}\right)$ and $L^{D_{u}^{cj2}}\left(R_{U_{k}}\vee_{p}LSL_{u}^{cj}\right)$ represent the total flight distances of $R_{U_{k}}$ after inserting the candidate depot combination ($LSL_{u}^{cj}$) and $D_{u}^{cj1}$ and $D_{u}^{cj2}$ indicate which depot in $LSL_{u}^{cj}$ is replaced with $D_{z}$.
2. Insertion at the end of $R_{U_{k}}$: The insertion position (*p*) is shown in Figure 5b. We use $D_{z}$ to denote the last landing depot in $R_{U_{k}}$, that is, $D_{z}=D_{u}^{k\delta 2}$. If $D_{u}^{cj1}=D_{u}^{cj2}=D_{z}$, then $LSL_{u}^{cj}$ can be directly inserted into position *p*. The total distance of the new $R_{U_{k}}$ can be expressed as follows:
  (17) $$L\left(R_{U_{k}}\vee_{p}LSL_{u}^{cj}\right)\;=\;L\left(R_{U_{k}}\right)+L\left(LSL_{u}^{cj}\right).$$
  If $D_{u}^{cj1}=D_{z}$ and $D_{u}^{cj1}\neq D_{u}^{cj2}$, then we insert $LSL_{u}^{cj}$ directly into position *p* with $D_{u}^{cj1}$ as the take-off depot. The total distance of the new $R_{U_{k}}$ can be obtained from Formula (17). When $D_{u}^{cj2}=D_{z}$ and $D_{u}^{cj1}\neq D_{u}^{cj2}$, the same principle applies. We insert $LSL_{u}^{cj}$ directly into position *p* with $D_{u}^{cj2}$ as the take-off depot.
  If $D_{u}^{cj1}\neq D_{z}$ and $D_{u}^{cj2}\neq D_{z}$, $D_{u}^{cj1}$ and $D_{u}^{cj2}$ may be the same or different. In this case, it is necessary to calculate the total distances of the new $R_{U_{k}}$ after replacing $D_{u}^{cj1}$ with $D_{z}$ and replacing $D_{u}^{cj2}$ with $D_{z}$ when inserting $LSL_{u}^{cj}$ into position *p*. They can be expressed as follows:
  (18) $$L^{D_{u}^{cj1}}\left(R_{U_{k}}\vee_{p}LSL_{u}^{cj}\right)\;=\;L\left(R_{U_{k}}\right)+L^{D_{z}}\left(LSL_{u}^{cj},D_{u}^{cj1}\right),$$
  (19) $$L^{D_{u}^{cj2}}\left(R_{U_{k}}\vee_{p}LSL_{u}^{cj}\right)\;=\;L\left(R_{U_{k}}\right)+L^{D_{z}}\left(LSL_{u}^{cj},D_{u}^{cj2}\right),$$
  After that, we use Formula (16) to select the minimum total distance as the total flight distance of $R_{U_{k}}$ after inserting $LSL_{u}^{cj}$ at position *p*.
3. Insertion between two depot–task set–depot combinations of $R_{U_{k}}$: The insertion position (*p*) is shown in Figure 5c. We use $D_{z}$ to denote the landing depot of the previous combination and the take-off depot of the next combination at position *p*, that is, $D_{z}=D_{u}^{k\alpha 2}=D_{u}^{k\beta 1}$. If $D_{u}^{cj1}=D_{u}^{cj2}=D_{z}$, then $LSL_{u}^{cj}$ can be directly inserted into position *p*. The total distance of the new $R_{U_{k}}$ can be obtained from Formula (17). If $D_{u}^{cj1}=D_{z}$ and $D_{u}^{cj1}\neq D_{u}^{cj2}$, we insert $LSL_{u}^{cj}$ into position *p* with $D_{u}^{cj1}$ as the take-off depot and, at the same time, replace the take-off depot of $LSL_{u}^{k\beta }$ with $D_{u}^{cj2}$. When $D_{u}^{cj2}=D_{z}$ and $D_{u}^{cj1}\neq D_{u}^{cj2}$, the same principle applies. We insert $LSL_{u}^{cj}$ into position *p* with $D_{u}^{cj2}$ as the take-off depot and, at the same time, replace the take-off depot of $LSL_{u}^{k\beta }$ with $D_{u}^{cj1}$. Then, the total length of the new $R_{U_{k}}$ can be obtained from Formula (13). If $D_{u}^{cj1}\neq D_{z}$ and $D_{u}^{cj2}\neq D_{z}$, where $D_{u}^{cj1}$ and $D_{u}^{cj2}$ may be the same or different, the method of adjustment after insertion is the same as that under the same condition when inserting at the beginning of $R_{U_{k}}$. Therefore, the total distance of the new $R_{U_{k}}$ can be obtained from Formula (16).

For the above three different situations, when adjusting the depot–task set–depot combinations, if the total distance of the adjusted depot–task set–depot combinations exceeds $L_{max}$ and the total load exceeds $M_{max}^{u}$, the currently adjusted route does not meet the energy consumption limit of the UAV and cannot be inserted into position *p*. When making adjustments to trucks, there is no need to consider the distance limit. After judging each insertion point in $R_{U_{k}}$, we can obtain the insertion point that results in the minimum total distance of the new path after insertion. This insertion point *p* serves as the optimal insertion point of $R_{U_{k}}$, and at the same time, we can obtain the corresponding new $R_{U_{k}}$ and its total distance. Therefore, we have the following:

(20) $$L\left(R_{U_{k}}\vee LSL_{u}^{cj}\right)=\min_{p=1}^{|R_{U_{k}}|+1}\left\{L\left(R_{U_{k}}\vee_{p}LSL_{u}^{cj}\right)\right\}.$$

## 5. Simulation Results

In this section, to verify whether the task-planning algorithm based on the minimum-cost, maximum-flow model, the resource tree, and the insertion method proposed in Section 4 is feasible and effective, we conduct simulation experiments using Matlab 2018a. To test the task allocation algorithm for the truck-independent UAV system, we design a task scenario as follows. The task area is set to be 1000 m × 1000 m. There are 3 depots, 2 independent UAVs, 2 trucks, and 15 delivery task points in this area. The task points are divided into 4 overweight task points and 11 non-overweight task points. The coordinate values of the task points and depots are randomly generated within the task area, and it is assumed that all location information is known in advance.

**Figure 6:**
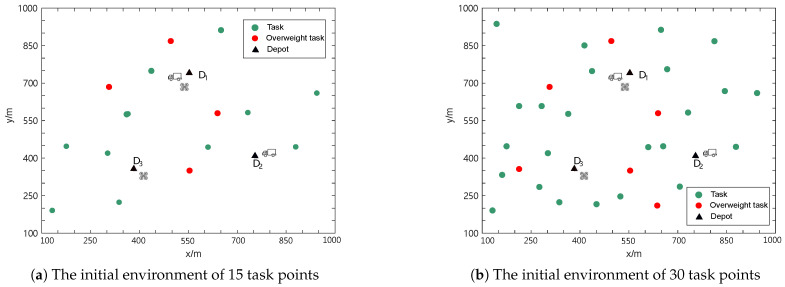
Diagrams of initial environments.

The initial docking depots of UAVs $U_{1}$ and $U_{2}$ are $D_{1}$ and $D_{3}$, respectively, and the initial docking depots of trucks $T_{1}$ and $T_{2}$ are $D_{1}$ and $D_{2}$, respectively. The initial environment of this scenario is shown in Figure 6a. In addition, the maximum flight distance of the UAVs is specified as $L_{max}$ = 600 m, the maximum load capacity of the UAVs is $M_{max}^{u}$ = 15 kg, and the maximum load of the trucks is $M_{max}^{t}$ = 50 kg.

To further test our algorithm, we design another more complex scenario to verify the minimum-cost, maximum-flow and resource tree-based logistics planning algorithm (MRTP) and compare it with the greedy algorithm-based logistics planning algorithm (GTP). In the other scenario, the task area contains 30 delivery task points, which are divided into 6 overweight task points and 24 non-overweight task points. The position of each task point is randomly generated. Other parameters are the same as those in the previous scenario.

**Figure 7:**
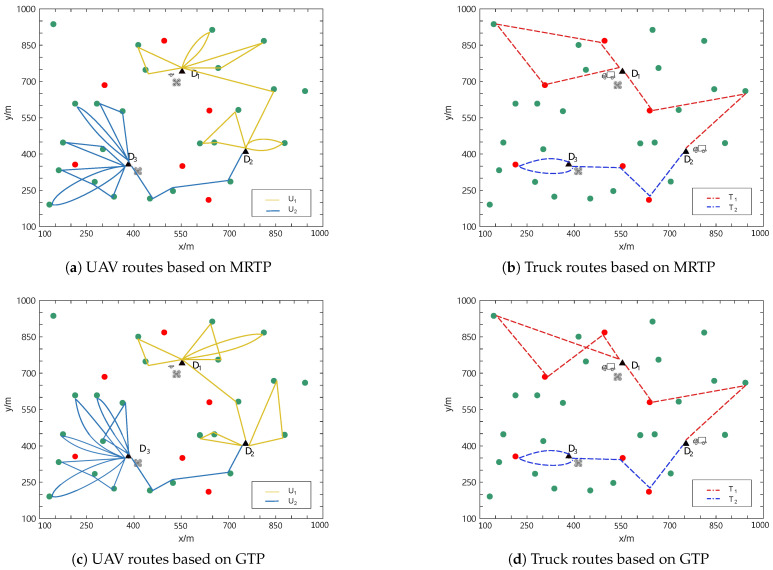
Task allocation results of MRTP and GTP with 30 task points.

**Figure 8:**
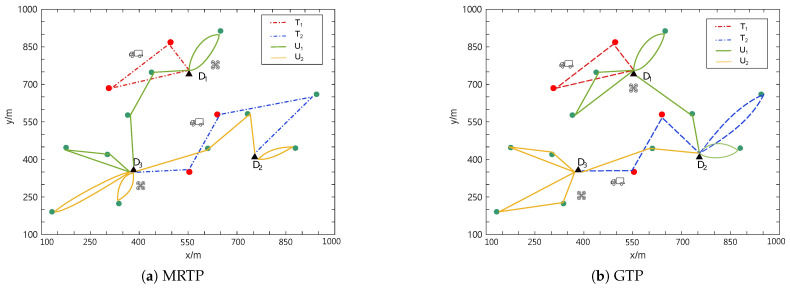
Task allocation results of MRTP and GTP with 15 task points.

The task allocation results obtained for the two scenarios using MRTP and GTP are shown in Figure 7 and Figure 8, respectively, where the routes of UAVs are represented by solid lines and the routes of trucks are represented by dashed lines.

As an example, in Figure 8a, UAV $U_{1}$ takes off from the initial docking depot ($D_{1}$) and returns to $D_{1}$ after completing the first flight mission. After replenishment, it takes off from depot $D_{1}$ again and heads for depot $D_{3}$, performing delivery tasks along the way. After landing at $D_{3}$, it replenishes again. After the replenishment is completed, it takes off again, finishes the delivery tasks, and returns to depot $D_{3}$.

The task allocation results of MRTP and GTP in different scenarios are shown in Table 2. It can be seen that in both scenarios, the total energy consumption obtained by MRTP is lower than that of GTP. Specifically, under different numbers of task points, MRTP achieves reductions in energy consumption of 11.53% and 9.15%. These results verify the feasibility and effectiveness of the proposed algorithm.

## 6. Conclusions

This paper focuses on the logistics task-planning problem for unmanned aerial vehicles (UAVs) in wide-area scenarios. Considering the load and energy limitations of UAVs, as well as the load constraints of trucks, a parcel delivery system based on a truck-independent UAV system is proposed. To address factors such as the UAVs’ load and energy capacities and the trucks’ load constraints, corresponding objective functions and constraints are defined, leading to the establishment of a mathematical model for this problem. First, a task-planning algorithm based on the minimum-cost, maximum-flow method is used to divide all task points into multiple depot–task set–depot combinations. Overweight task points and those beyond the UAV delivery range are assigned to trucks, while the rest are handled by UAVs. Next, an algorithm based on a resource tree is designed to construct the initial paths for UAVs and trucks. Finally, an insertion algorithm is proposed to adjust the initial paths of UAVs and trucks according to the candidate depot–task set–depot combinations. Experimental results demonstrate that this algorithm effectively optimizes the system’s energy consumption. However, the method’s scalability is limited when the number of UAVs and trucks increases or task scenarios change. Future work will address these limitations by exploring adaptive algorithms and enhancing system flexibility to accommodate more complex operational conditions.

## Figures and Tables

**Table 1:** Summary of important notations.

Notation	Definition
*Q*, *D*, *q*	Number, set, and index of depots, respectively
*F*	Numbers of customers
*S*, *f*, *M*	Set, index, and package-weight set of task points, respecitvely
*E*, *T*, *e*	Number, set, and index of trucks, respectively
*N*, *U*, *n*	Number, set, and index of UAVs, respectively
$L_{max}$	Maximum flight distance of UAVs
$M_{max}^{u}$	Maximum load capacity of UAVs
$M_{max}^{t}$	Maximum load capacity of the trucks
$R_{U}$, $U_{k}$	Set and index of task sets for *N* UAVs, respectively
$R_{T}$	Set of task sets for *E* trucks
$R_{T_{k}}$	Route arranged for trucks
$L_{R_{U_{ki}}}$	The flight distance of UAV $U_{k}$
$L_{R_{T_{kj}}}$	The travel distance of truck $T_{k}$
$W_{U}$	Total energy consumption of UAVs
$W_{T}$	Total energy consumption of trucks
$G_{U}$	Flow-cost network graph
Num	Number of task points
LSL	The depot–task set–depot combinations
DD	Set of depot combinations
V,E,C,ω	Set of points, edges of $G_{U}$, capacity of each edge, and total cost of each edge, respectively

**Table 2:** Task allocation results of MRTP and GTP.

Method	Number of UAVs	Number of Trucks	Number of Task Points	Total Energy Consumption
MRTP	2	2	15	6.9
GTP	2	2	15	7.8
MRTP	2	2	30	27.8
GTP	2	2	30	30.6

## Data Availability

The data used to support the findings of this study are available from the corresponding author upon request.
